# Live and Heat-Inactivated *Streptococcus thermophilus* MN-ZLW-002 Mediate the Gut–Brain Axis, Alleviating Cognitive Dysfunction in APP/PS1 Mice

**DOI:** 10.3390/nu16060844

**Published:** 2024-03-15

**Authors:** Yujie Zhang, Yimei Wang, Zhimo Zhou, Yang Yang, Jincheng Zhao, Xiaohong Kang, Zhouyong Li, Xi Shen, Fang He, Ruyue Cheng

**Affiliations:** 1 Department of Nutrition and Food Hygiene, West China School of Public Health and West China Fourth Hospital, Sichuan University, Chengdu 610041, China; yujiezhang1031@163.com (Y.Z.); may_wang12@126.com (Y.W.); gwrzzm@163.com (Z.Z.); yywishyoubehappy@stu.scu.edu.cn (Y.Y.); jinchengz348@gmail.com (J.Z.); hxgwshenxi@sina.com (X.S.); 2 Global R&D Innovation Center, Inner Mongolia Mengniu Dairy (Group) Co., Ltd., Hohhot 011050, China; kangxiaohong@mengniu.cn (X.K.); lizhouyong@mengniu.cn (Z.L.)

**Keywords:** Alzheimer’s disease, gut microbiota, probiotics, postbiotics

## Abstract

Research on regulating brain functions with probiotics and postbiotics through the gut–brain axis is attracting attention, offering the possibility of adjuvant therapy for Alzheimer’s disease (AD). Three-month-old male APP/PS1 mice were gavaged with live and heat-inactivated *S. thermophilus* MN-002 for three months. This study demonstrated that live and heat-inactivated *S. thermophilus* MN-002 improved cognitive dysfunctions in APP/PS1 mice, especially in spatial memory. However, the main effects of live *S. thermophilus* MN-002 directly altered the intestinal microbiota composition and increased serum IL-1β and IL-6. Heat-inactivated *S. thermophilus* MN-002 increased colonic propionic acid levels and enhanced the hippocampus’s antioxidant capacity. Furthermore, the changes were more obvious in the high-dose group, such as astrogliosis in the hippocampus. These results indicate that different forms and doses of the same strain, *S. thermophilus* MN-002, can partly improve cognitive functions in AD model mice via the gut–brain axis.

## 1. Introduction

Alzheimer’s disease (AD) is a neurodegenerative disease featuring rapid cognitive impairment. It is the most common cause of dementia, manifesting as loss and difficulties in memory, thinking, and language skills [1]. More than 50 million people were living with AD worldwide in 2018, and the number will increase to 100 million by 2050 [2]. AD has a long prodromal stage and median survival period; therefore, its burden on individuals and society cannot be ignored. Extensive research into preventing and treating AD has important implications for public health and clinical medicine.

The human gut contains several microorganisms involved in the metabolic processes of the body, which can be influenced by various factors, including delivery and food and drug intake [3,4]. The microbiota functions as an intestinal barrier and regulates the distal organs. This phenomenon is observed in several different organs, with distinct gut–organ axes including the gut–brain, gut–skin, and gut–lung axes [5,6,7]. In the case of the gut–brain axis, for example, studies have shown significant differences between the gut microbiota of patients with AD and healthy controls [8]. In some in vivo studies, we demonstrated that interventions for gut microbiota reduced neurological symptoms, particularly some additional probiotic supplements that improved cognitive functions in patients with AD [9,10,11]. There is a strong link between gut microbiota composition and cognitive functions. Regulators enable a change in gut microbiota and positively affect cognitive functions in AD via the gut–brain axis.

Probiotics, postbiotics, and prebiotics are the most widely used regulators of gut microbiota composition [12]. Probiotics are active microorganisms that have health-promoting effects on the host when used appropriately [13]. Postbiotics are preparations of inactivated microorganisms and/or their components that confer health benefits on the host [14]. A study showed that *Lactobacillus plantarum* MTCC1325 could improve cognitive functions in AD model mice [15]. Another study showed that *Bifidobacterium bifidum* BGN4 and *Bifidobacterium longum* BORI improved cognitive decline in older adults with cognitive impairment [16]. However, the health-promoting effects of probiotics are strain-specific [17]. *Streptococcus thermophilus* MN-ZLW-002 (*S. thermophilus* MN-002), an isolated strain with high extracellular polysaccharide production from traditional fermented dairy products in the Gannan region, was used in this study. The strain has a role in inhibiting pre-adipocyte differentiation and regulating metabolism [18,19]. Our previous study demonstrated that heat-inactivated *Lacticaseibacillus paracasei* N1115 improved brain functions in mice with damage from an antibiotic cocktail [20]. These results suggest that probiotics and postbiotics benefit gut microbiota and cognitive functions. Therefore, the effects of gut microbiota regulators are related not only to the strain but also to whether the bacteria are live or heat-inactivated. However, only a few studies have reported the differences in the cognitive-function-promoting effects between live and heat-inactivated bacteria in the same strain and whether these effects could last even after the intervention.

Therefore, this study investigated whether live and heat-inactivated postbiotic elements of *S. thermophilus* MN-002 continuously ameliorate effects on cognitive impairment in AD animal models and whether there are differences underlying the mechanisms of action.

## 2. Materials and Methods

### 2.1. Animals

Seventy-five male APPswe/PS1Δe9 (APP/PS1) mice aged three months were used as the model and experimental groups, and fifteen age-matched wild-type mice were used as the control group. The APP/PS1 mice were divided into the model group, the low-dose live probiotics treatment (AL) group, the high-dose live probiotics treatment (AH) group, the low-dose heat-inactivated probiotics treatment (IL) group, and the high-dose heat-inactivated probiotics treatment (IH) group. All the mice were housed in a specific-pathogen-free facility. They were allowed free access to food and water ad libitum. The animals were maintained on a 12:12 light–dark cycle (lights on at 08:00, lights off at 20:00) in a room with controlled temperature (21.5 °C ± 1 °C) and air humidity (55% ± 5%).

**Figure 1:**
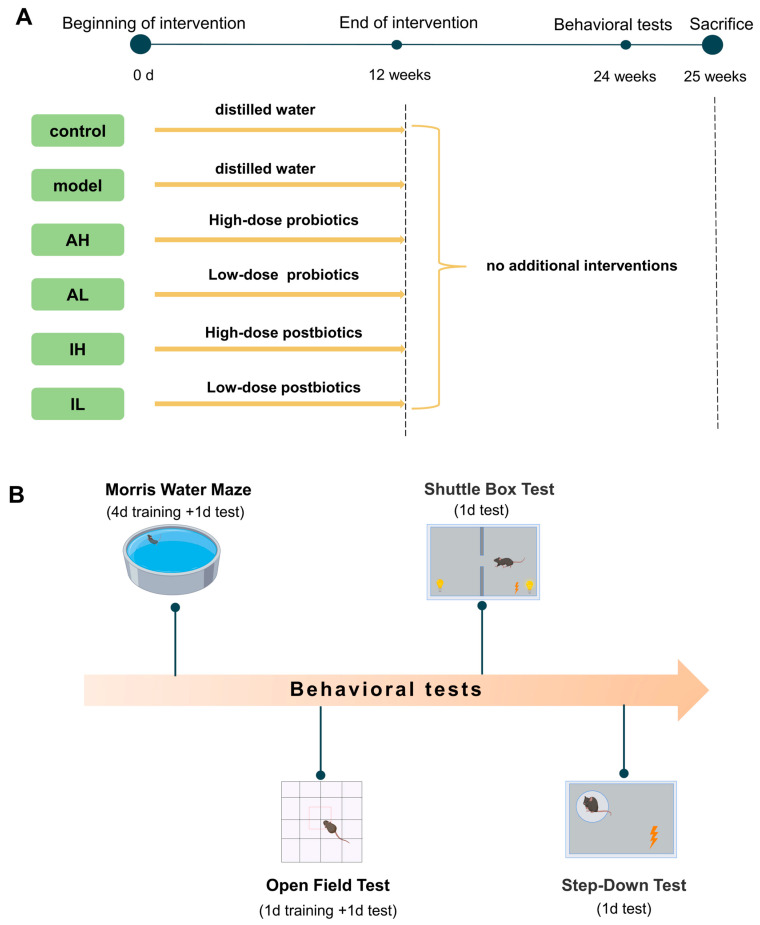
Protocol of the study. (**A**). Experimental groupings and interventions. (**B**). Content and sequence of behavioral tests.

All the mice received interventions for 12 weeks. After the interventions, the mice were fed for another 12 weeks without any other interventions. Behavioral tests started at 25 weeks, including the Morris water maze test, open field test, shuttle box test, and step-down test. At the end of the behavioral tests, the mice were sacrificed, and samples were collected. The experimental design was demonstrated in Figure 1. The experiment was performed at the Public Health and Preventive Medicine Provincial Experiment Teaching Center at Sichuan University and the Food Safety Monitoring and Risk Assessment Key Laboratory of Sichuan Province. At the end of the experiment, the euthanasia process for the mice consisted of anesthesia with ethyl ether using an anesthesia machine, collection of blood from the abdominal aorta blood under complete anesthesia, and finally decapitation.

### 2.2. Probiotic Treatment

The experimental groups received *S. thermophilus* MN-002 as the intervention strain. The low-dose AL and IL groups were gavaged with 8.33 × 10^9^ CFU/kg.bw/d, and the high-dose AH and IH groups were gavaged with 1.67 × 10^10^ CFU/kg.bw/d. To prepare this strain for the inactivated probiotic groups, it was dissolved in sterile saline and heated at 65 °C for 2 h. The control and model groups were gavaged with distilled water. The gavage volume given to each mouse was 200 μL/d. *S. thermophilus* MN-002 was kindly supplied by Mengniu Dairy (Group) Co., Ltd. (Hohhot, China).

### 2.3. Morris Water Maze Test (MWM)

The MWM consisted of four equally divided right-angled sector quadrants forming a circular area of 80 cm in diameter. The platform was placed 10 cm from the outer wall of the Morris water maze. The water used in the test was maintained at 21–22 °C. The duration of the experiment was four training days and one test day. Each experiment lasted 60 s; experiments were conducted four times a day during the training period and only once on the test day. The latency was defined by the first time the mice passed the platform’s position.

### 2.4. Open Field Test (OFT)

The equipment for the open field experiment consisted of a 60 cm square box with a probe above it. The bottom of the box was divided into nine square areas of the same size. Each mouse was tested for 5 min, and the trajectory and rest time spent in different areas were recorded. A solution of approximately 75% alcohol was used to clean up the mouse traces and odors after each trial to avoid interference with the experimental results.

### 2.5. Shuttle Box Test

The equipment for the shuttle box test consisted of two identical-sized chambers and a middle door linking the two chambers. Each chamber had a light bulb at the top and an electrically accessible wire mesh at the bottom. After the mice entered the chamber, the door was opened to connect the left and right chambers. Before the test, mice were exposed to the apparatus for a 30 s habituation period. At the beginning of the experiment, the light was illuminated where the mice were located as a conditioned stimulus, and 3 s later, the bottom of the area was electrified to give an unconditioned stimulus. Escape from the area after stimulation was considered to be one escape. One conditioned stimulus and one unconditioned stimulus constituted a cycle, with a 10 s interval between cycles, and the test was conducted for 5 min (15 cycles). The numbers of escapes after a conditioned stimulus and an unconditioned stimulus were recorded.

### 2.6. Step-Down Test

The equipment for the step-down test consisted of eight equal-sized chambers, each with an insulated platform. The chambers had electrically accessible wire mesh at the bottom. Each mouse had a 1 min habituation period on the first day, and the chambers were electrified for 30 s. After finishing those stages, each mouse was tested in each electrified chamber for 5 min. After 24 h, the experiment was repeated. The number of times the mice jumped off the platform was recorded as the number of errors.

### 2.7. Enzyme-Linked Immunosorbent Assay (ELISA)

The mice were sacrificed at 25 weeks. Serum samples were collected to measure the levels of interleukin-1β (IL-1β), interleukin-6 (IL-6), tumor necrosis factor-α (TNF-α), lipopolysaccharide (LPS), and C-reactive protein (CRP). ELISA kits (R&D Systems Inc., Minneapolis, MN, USA, and CUSABIO Biotechnology Co., Ltd., Wuhan, China) were used for the experimental assays, and the operations were carried out strictly following the manufacturer’s instructions.

Hippocampal brain tissues were collected for biochemical analysis. The samples were centrifuged at 5000× *g* for 10 min after homogenization with PBS (1:9, weight/volume). The glutathione (GSH), glutathione peroxidase (GSH-Px), superoxide dismutase (SOD), and catalase (CAT) activity and the malondialdehyde (MDA) levels were measured with corresponding kits (Jonln Biotechnology Co., Ltd., Shanghai, China) according to the manufacturer’s instructions.

### 2.8. Reverse Transcription-Quantitative Polymerase Chain Reaction (RT-qPCR)

Hippocampal tissues were analyzed using RT-qPCR to measure the mRNA expression levels of brain-derived neurotrophic factor (BDNF), TNF-α, and IL-6. Ileal tissues were collected to measure the mRNA expression levels of occludin and ZO-1. Total RNA was extracted using an animal tissue total RNA extraction kit (Foregene Biotechnology Co., Ltd., Chengdu, China). Reverse transcription was performed using the iScriptTM gDNA Clear cDNA Synthesis Kit (Bio-Rad Laboratories, Berkeley, CA, USA). The reverse transcription reaction system consisted of 4 μL 5× iScript reaction mix, 1 μL iscript Reverse Transcriptase, 14 μL ddH2O, and 1 μL template RNA.

The amplification reaction system consisted of 5 μL of SsoFast EvaGreen supermix, 0.3 μL of forward primer, 0.3 μL of reverse primer, and 1 μL of template cDNA. The PCR cycling conditions were as follows: 95 °C for 30 s, 95 °C for 5 s, and Tm °C 5 s for 40 cycles. The dissolution curve was analyzed from 65 °C to 95 °C. The primer sequences are listed in Table 1.

### 2.9. 16S rRNA-Encoding Gene Sequencing and Bioinformatics Analysis

Fresh stool samples were collected for 16S rRNA sequencing. The total DNA was extracted using the TIANamo Stool DNA kit (Tiangen Biotech Co., Ltd., Beijing, China). Specific primers amplified the 16S rRNA gene’s V4 region. The reaction system for PCR included 15 µL of Phusion^®^ High-Fidelity PCR Master Mix (Thermo Fisher Scientific Inc., Shanghai, China), 0.2 µM of forward and reverse primers, and about 10 ng of template DNA. The cycling conditions were as follows; 98 °C for 1 min; 30 cycles of denaturation at 98 °C for 10 s, annealing at 50 °C for 30 s, and elongation at 72 °C for 30 s; and 72 °C for 5 min. Equal volumes of PCR products and SYBR green loading buffer were mixed, and electrophoresis was performed on 2% agarose gel for quantification. A DNA PCR-Free Sample Preparation Kit (Illumina. Inc., San Diego, CA, USA) was used to generate sequencing libraries. A Qubit@ 2.0 Fluorometer (Thermo Scientific. Co., Ltd., Waltham, MA, USA) and an Agilent Bioanalyzer 2100 system were used to assess the quality of the libraries. The library was sequenced on the Illumina NovaSeq platform (NovaSeq 6000), and 250 bp paired-end reads were generated. The sliding window method was employed to filter bases and choose effective sequences. The operational taxonomic units (OTUs) were analyzed. OTUs are defined by sequences with ≥97% similarity.

α diversity was used to represent the complexity of species diversity of the samples. It can be described by the Chao1, Shannon, Simpson, and ACE indices. The study calculated indices with QIIME (Version 1.7.0) and displayed them with R software (Version 2.15.3). β diversity was used to represent the species complexity difference between the samples using QIIME software (Version 1.9.1). Cluster analysis was preceded by principal coordinates analysis (PCoA), which was applied to reduce the dimensions of the original variables using the FactoMineR package and the ggplot2 package in R software (Version 2.15.3).

### 2.10. Immunofluorescence and Immunohistochemistry

The hippocampal tissues were fixed in 4% paraformaldehyde for 48 h. After fixation, the frozen sections were analyzed. The samples were permeabilized using 0.3% Triton X-100 for 5 min and blocked using 5% serum for 1 h at room temperature. The samples were incubated with primary antibodies against IBA-1 and GFAP overnight at 4 °C and incubated with secondary antibodies for 2 h at room temperature. The immunofluorescence intensity was observed with a fluorescence microscope.

The brain samples were fixed in 4% paraformaldehyde for 48 h. After fixation, the samples were cut into paraffin-embedded slices. The slices were sequentially placed in dewaxing solution I for 10 min, dewaxing solution II for 10 min, anhydrous ethanol III solution for 10 min, anhydrous ethanol I for 5 min, and anhydrous ethanol II and III for 5 min each, then washed with distilled water for 5 min. The tissues were covered uniformly by dropwise addition of 3% BSA on the histochemical sections and covered at room temperature for 30 min. The samples were incubated with anti-Aβ40 primary antibodies overnight at 4 °C and incubated with secondary antibodies for 50 min at room temperature. Fresh DAB solution was added dropwise on the slices; the cell nuclei were restained with hematoxylin and observed under a microscope.

### 2.11. Detection of Short-Chain Fatty Acids in Colonic Contents

The colonic contents (100 mg) were mixed and homogenized in 400 μL of ether, 100 μL of 4-methyl valeric acid, and 100 μL of 15% phosphoric acid. The mixture was centrifuged at 13,400× *g* for 10 min at 4 °C. The supernatant was aspirated for testing on an Agilent 7890A.

### 2.12. Statistical Analysis

All statistical analyses were performed using SPSS 23.0 (SPSS, Inc., Armonk, NY, USA). The data are expressed as the mean ± SEM. The normally distributed data were analyzed with one-way ANOVA, and Tukey’s test was used for comparing the groups. The data that did not conform to the normal distribution were analyzed with Kruskal–Wallis one-way ANOVA. The training latency in the MWM was analyzed by three-way ANOVA. The statistical results were considered significant at *p* < 0.05.

## 3. Results

### 3.1. Changes in Spatial Memory, Anxiety, and Short-Term Memory

**Figure 2:**
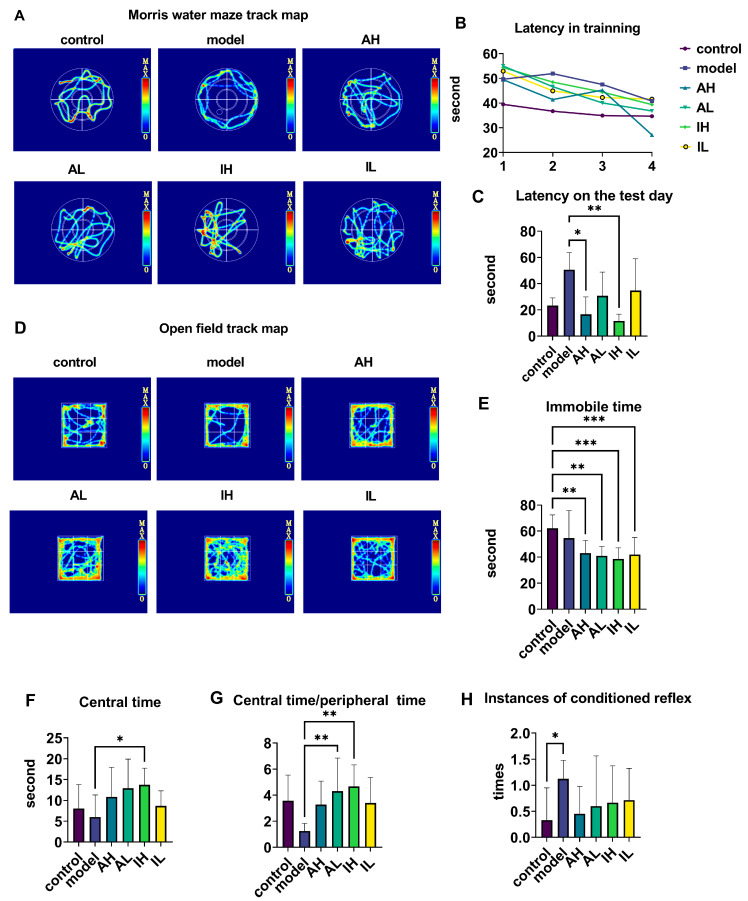
The effect of different doses of live and heat-inactivated *S. thermophilus* MN-002 on behavioral indicators. (**A**–**C**) are the track heat map, latency on the training day, and latency on the test day in the Morris water maze (MWM). (**D**–**G**) are the track heat map, immobile time, centeral time, and ratio of centeral time to peripheral time in the Open field test (OFT). (**H**) shows the number of instances of the conditioned reflex in the step-down test. * *p* < 0.05, ** *p* < 0.01, *** *p* < 0.001.

The navigation-path heat maps of the MWM showed that the activity was most intensive in the quadrant where the platform was located and in the adjacent quadrant for all groups except the model group (Figure 2A). During the training period, the latency of the APP/PS1 mice was longer than that of the control mice; however, at the end of the training, all groups reached the platform and had shorter latency than at the beginning (Figure 2B). On the test day, compared with the model groups, the AH and IH groups had reduced latencies (Figure 2C).

The navigation path in the heat map of the OFT showed that the activity was more intensive in the central area than in the surroundings for all groups except the model group (Figure 2D). Compared with the control group, the AH, AL, IH, and IL groups had much shorter immobile times during the open field test (Figure 2E). Compared with the model group, the IH group spent a long time in the central area (Figure 2F). Compared with the model group, the ratio of central time to peripheral time of OFT was increased in the AL and IH groups (Figure 2G). Compared with the control group, the model group had an increase in conditioned reflexes (Figure 2H).

### 3.2. Changes in Hippocampal Deposits of Aβ40, Astrogliosis, and Microgliosis

**Figure 3:**
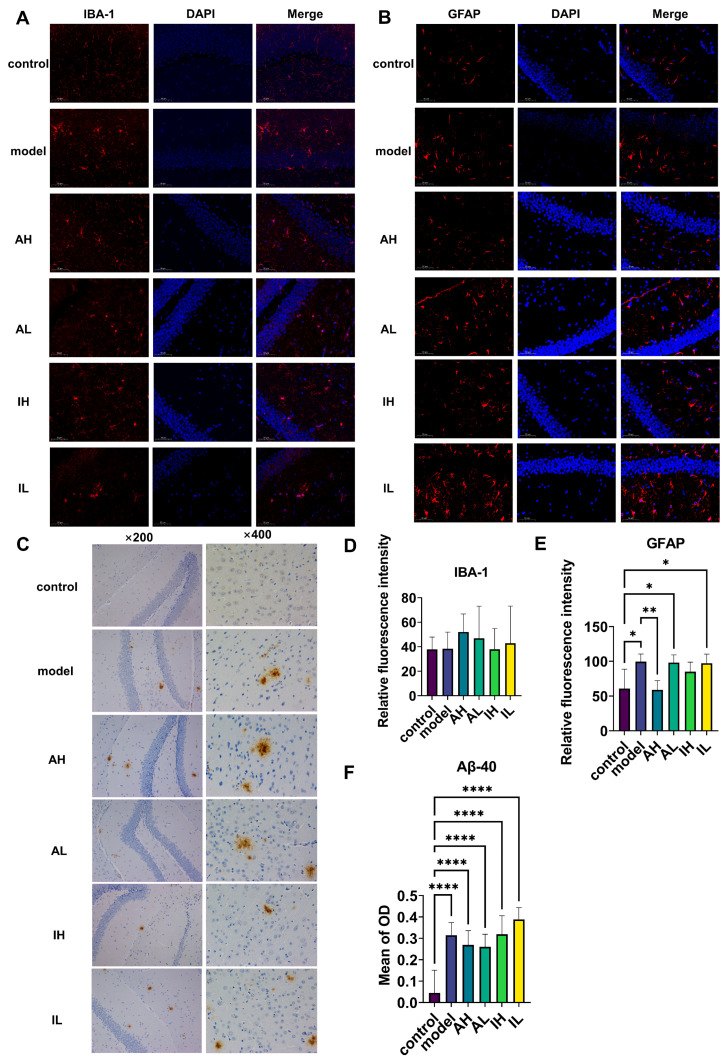
The effect of different doses of live and heat-inactivated *S. thermophilus* MN-002 on astrocytes, microglia, and amyloid-β 40 (Aβ-40). (**A**,**D**) show expression of ionized calcium binding adaptor molecule 1 (IBA-1) in microglia. (**B**,**E**) show expression of glial fibrillary acidic protein (GFAP) in astrocytes. (**C**,**F**) show deposition of Aβ-40 in the hippocampus. * *p* < 0.05, ** *p* < 0.01, **** *p* < 0.0001.

There was no significant difference observed in IBA-1 (Figure 3A,D). Compared with the control group, the model, AL, and IL groups had significantly increased levels of astrogliosis. In contrast, compared to the model group, the AH group had a significantly reduced level of astrogliosis (Figure 3B,E). Compared with the control group, all the APP/PS1 mice had increased deposition of Aβ40; however, there was no difference between the different groups of APP/PS1 mice (Figure 3C,F).

### 3.3. Alterations in Gut Microbiota and Their Metabolites

**Figure 4:**
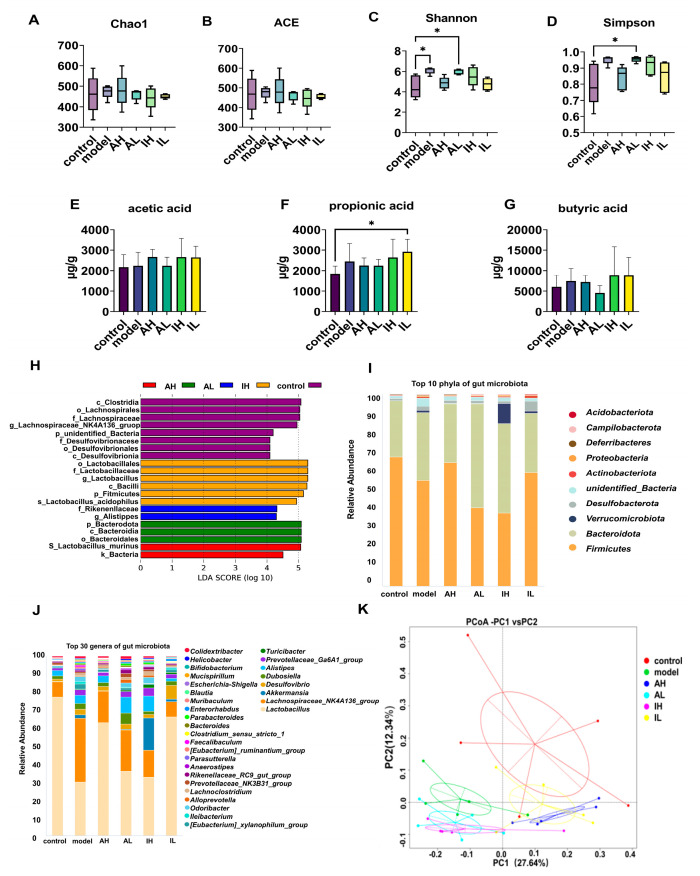
The effect of different doses of live and heat-inactivated *S. thermophilus* MN-002 on gut microbiota and their short-chain fatty acids. (**A**–**D**) are indexes of α diversity analysis. (**E**–**G**) are the short-chain fatty acid levels of the cecal contents. (**H**) is the effect-size analysis using Linear Discriminant Analysis (LDA). (**I**,**J**) are the top 10 phyla and top 30 genera of gut microbiota. (**K**) is the principal coordinates analysis (PCoA) of gut microbiota. * *p* < 0.05.

No significant difference was observed in the ACE or Chao1 indexes (Figure 4A,B). Compared with the control group, the model and AL groups had increased Shannon indexes (Figure 4C). Compared with the control group, the AL group had an increased Simpson index (Figure 4D).

The effect-size analysis using LDA showed that the biomarkers of different groups were not similar, and the biomarkers differed across groups at multiple levels. The biomarkers were *Lactobacillales*, *Lactobacillaceae*, and *Lactobacillus* in the control group; *Clostridia* in the model group; *Lactobacillus_murinus* in the AH group; *Bacteroidota* in the AL group; and *Rikenellaceae* in the IH group (Figure 4H).

The composition of the gut microbiota was significantly altered. At the phylum level, *Actinobacteriota* in the IL group, *Bacteroidota* and *Campilobacterota* in the AL group, *Deferribacteres* and *Verrucomicrobiota* in the model group, and *Verrucomicrobiota* in the IH group were more abundant than in the control group. *Firmicutes* in the AL and IH groups were less abundant than in the control group. *Bacteroidota* in the AL and IL groups was more abundant than in the model group. *Deferribacteres* in AH, AL, and IH groups were more abundant than in the model group, while the abundance of that taxon in the IL group was lower than in the model group (Table 2, Figure 4I).

At the genus level, *Tuzzerella* and *Helicobacter* in the AL group; *Akkermansia* in the model and IH groups; *Bifidobacterium*, *Ileibacterium*, *Negativibacillus*, and *Intestinimonas* in the model and AL groups; *Blautia* in the model, AH and IH groups; *Enterorhabdus* in the IL group; *Monoglobus* and *Parasutterella* in the model group; *Oscillibacter* in the AH group, *Parvibacter* in the AL and IL groups; *Streptococcus* in the IH and IL groups; and *Turicibacter* and *Mucispirillum* in the model and IH groups were more abundant than in the control group. *Lactobacillus* in the model, AL, and IH groups; *Odoribacter* in the model group; and *Tuzzerella* in the IH group were less abundant than in the control group. *Mucispirillum* in the AL and IH groups was more abundant than in the model group. *Bifidobacterium* in the AH, AL, and IH groups; *Mucispirillum* in the AL, IH, and IL groups; *Oscillibacter* in the IL group; *Parasutterella* in the AH and IL groups; and *Tuzzerella* in the IL group were less abundant than in the model group (Table 3, Figure 4J).

The content of short-chain fatty acids, the primary metabolite of the gut microbiota, varies as the intestinal microbiota changes. The IL group had significantly higher propionic acid levels than the control group (Figure 4F). One-way ANOVA revealed no significant differences in the levels of acetic acid or butyric acid among the groups (Figure 4E,G).

According to PCoA based on the weighted UniFrac distance, there were significant differences in the gut microbiota composition in different groups. PC1 could explain 27.64% of this variability, and PC2 could explain 12.34%. PC1 separated the control, AH, and IL groups from the other groups. In contrast, PC2 separated the groups of mice with APP/PS1 genes, indicating that the intervention with MN002 and the APP/PS1 genes could affect the gut microbiota (Figure 4K).

### 3.4. Changes in Serum Inflammatory Markers

**Figure 5:**
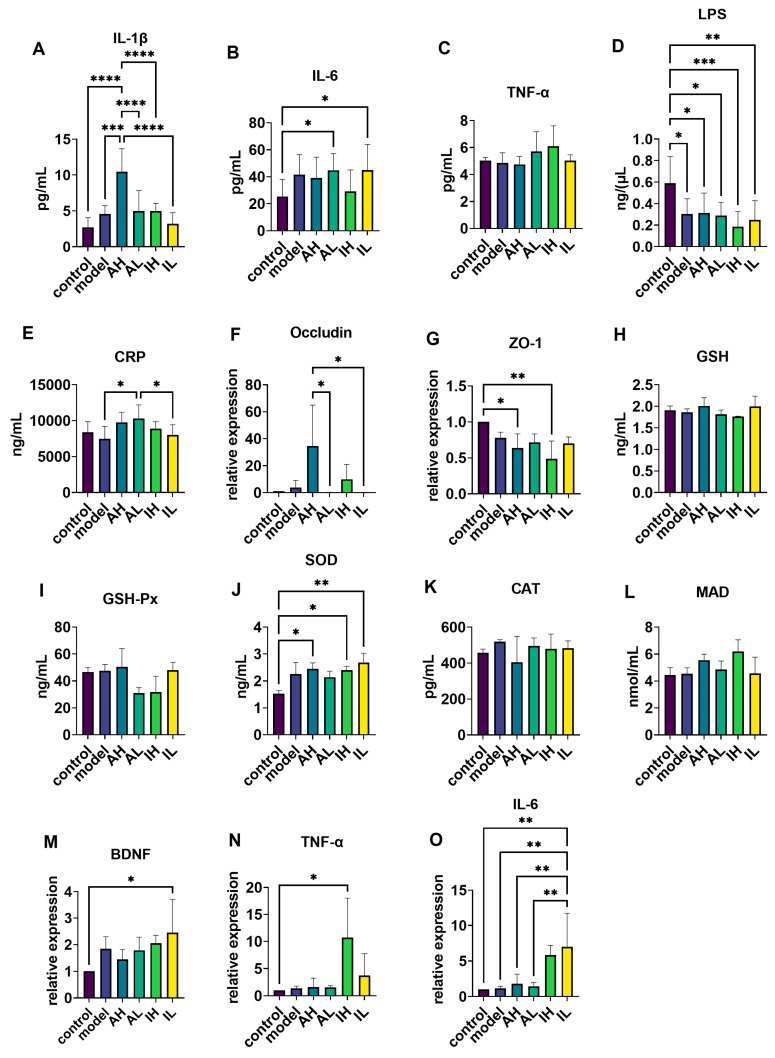
The effect of different doses of live and heat-inactivated *S. thermophilus* MN-002 on serum immune factors, intestinal tight junction proteins, antioxidant capacity, brain-derived neurotrophic factor (BDNF), and immunity-related factors. (**A**–**E**) were Interleukin—1β (IL-1β), Interleukin—6 (IL-6), tumor necrosis factor-α (TNF-α), Lipopolysaccharide (LPS), and C-reactive protein (CRP) levels of serum. (**F**,**G**) are the mRNA expression levels of tight junction proteins in the ileum. (**H**–**L**) are enzymes associated with antioxidant capacity in the hippocampus. (**M**–**O**) are the mRNA expression levels of BDNF, TNF-α, and IL-6 in the hippocampus. * *p* < 0.05, ** *p* < 0.01, *** *p* < 0.001, **** *p* < 0.0001.

Compared with the control and model groups, the AH group had significantly increased IL-1β levels in serum (Figure 5A). Compared with the control group, the AL and IL groups had significantly increased IL-6 levels in the serum (Figure 5B), while the model, AH, AL, IH, and IL groups had significantly decreased LPS levels in the serum (Figure 5D). Compared with the model and IL groups, the AL group had significantly increased CRP levels in the serum (Figure 5E). One-way ANOVA revealed no significant differences in the levels of TNF-α between the groups (Figure 5C).

### 3.5. Changes in the mRNA Expression of Ileum Tight Junction Proteins

Compared with the AH group, the AL and IL groups exhibited reduced mRNA expression levels of occludin (Figure 5F). Compared with the control group, the AH and IH groups demonstrated reduced mRNA expression levels of ZO-1 (Figure 5G).

### 3.6. Changes in Antioxidation-Related Enzymes, mRNA Levels of BDNF, and Inflammatory Marker Expression in the Hippocampus

Compared with the control group, the AH, IH, and IL groups had increased levels of SOD in the brain (Figure 5J). One-way ANOVA revealed no significant differences between the groups’ GSH, GSH-Px, CAT, or MAD levels (Figure 5H,I,K,L).

Compared with the control group, the IL group had increased levels of BDNF (Figure 5M). Compared with the control group, the IH group had increased levels of TNF-α (Figure 5N). Compared with the IL group, the control, model, AH, and AL groups had decreased levels of IL-6 (Figure 5O).

## 4. Discussion

According to the concept of the gut–brain axis, regulating the gut microbiota can modulate the host’s cognitive function. Several studies showed that certain probiotic strains could improve cognitive functions in AD model animals or patients; such strains include *Bifidobacterium shortum* A1, *Clostridium butyricum*, and *Akkermansia muciniphila* [10,21,22]. Other studies have shown that postbiotics can relieve anxiety and depression in vivo via the gut–brain axis [23,24]. Although studies have demonstrated the health-promoting effects of postbiotics on mood, there have been fewer studies on the promotion of central nervous system health, particularly the ameliorative effects of postbiotics on cognitive impairment in AD, and whether these effects could last after the intervention remains unclear. Herein, we used live and heat-inactivated *S. thermophilus* MN-002 as the tested strain to determine the potential continuous effects of probiotics and postbiotics in different doses on the cognitive function and the related mechanisms in AD model mice.

Our study demonstrated that live and heat-inactivated *S. thermophilus* MN-002 improved cognitive functions in mice with AD in the MWM test and that the inactivated high-dose postbiotic also relieved anxiety in AD mice, even after the interventions had been discontinued for three months. Although all the AD model mice exhibited high deposition of Aβ40 protein, high doses of live *S. thermophilus* MN-002 significantly reduced the number of astrocytes. These effects were especially pronounced in the high-dose group. Additionally, the composition of the gut microbiota at the phylum level was similar to that of the control group after using high doses of live *S. thermophilus* MN-002. Differences in composition between the two groups were not statistically significant. These results suggested that the effects of *S. thermophilus* MN-002 on the gut–brain axis are influenced by the status of bacteria.

The changes in the gut microbiota diversity correlated with results such as those of Bäuerl et al. They found that the main difference between the control and APP/PS1 mice was an increase in the Shannon index [25]. Many studies have concluded that health is strongly associated with higher gut microbiota diversity. A growing number of studies have shown an increase in microbiota diversity in patients with neurological disorders, such as depression and autism [26,27]. This suggests that the relationship between the gut microbiota and health needs to be studied with a greater focus on the composition and colonization of some specific bacteria rather than merely the diversity of gut microbiota. In our study, the main difference observed between the postbiotic and control groups was the proportion of *Verrucomicrobiota*. A significantly higher percentage of *Akkermansia* was observed in the IH group at the genus level. *Akkermansia* is a genus belonging to the phylum *Verrucomicrobiota*; it modulates the host’s immune functions; protects the intestinal mucosal barrier; and reduces the risk of metabolic diseases, such as obesity and diabetes [28,29,30]. On the other hand, the live *S. thermophilus* MN-002 groups had a closer microbiota community to the control group. Both of these effects were more potent in the higher-dose group than in the lower-dose group, as seen at the phylum and genus levels. These two different alterations suggest that the regulatory effects on gut microbiota by live *S. thermophilus* MN-002 are reflected in the changing compositions of the microbiota. In contrast, the alteration in heat-inactivated *S. thermophilus* MN-002 changes the proportion of specific bacteria, such as *Akkermansia*. The effects of higher doses of *S. thermophilus* MN-002 were more pronounced than the effects of lower doses.

These differences may be related to how live and heat-inactivated *S. thermophilus* MN-002 in different doses regulate the gut microbiota and cognitive functions. In addition to differences in the gut microbiota structure, changes in microbial metabolites also play an essential role in probiotics’ and postbiotics’ regulation of cognitive functions. It has been shown that propionate reduces motor impairment and dopamine neuron loss in Parkinson’s model mice [31]. In this study, we observed increased propionic acid after using low doses of heat-inactivated *S. thermophilus* MN-002. This may also be one of the ways in which it exert regulatory effects, but further in vitro experiments and microbial isolation and culture are needed to verify the specific key strains and mechanisms of action.

Regulating immune function is an essential bidirectional communication pathway for the gut–brain axis [32,33]. IL-1β and IL-6 are pro-inflammatory factors in common immune responses. Studies have shown that the intestinal barrier is incomplete in APP/PS1 mice compared to wild-type mice [34]. We, therefore, speculated that stimulatory effects on APP/PS1 mice with an incomplete intestinal barrier were exerted to some extent by live and heat-inactivated *S. thermophilus* MN-002, resulting in elevated serum IL-1β and IL-6 levels. CRP is a marker protein associated with inflammation and can be produced in response to the mediation of pro-inflammatory factors, such as IL-1β and IL-6 [35]. The AH group showed a significant increase in CRP, demonstrating that live *S. thermophilus* MN-002 has a more pronounced effect on the immune system.

In contrast, heat-inactivated *S. thermophilus* MN-002, which cannot colonize the gut, has less ability to modulate the immune system. Our results showed that high doses of live and heat-inactivated *S. thermophilus* MN-002 affected the expression of intestinal barrier tight junction proteins. Therefore, for hosts with incomplete intestinal mucosal barriers, high doses of probiotics or postbiotics do not necessarily result in more beneficial effects. Appropriate doses are essential for the health-promoting effects of probiotics and postbiotics.

We examined the changes in the antioxidant-related enzymes and immune-related indicators in the hippocampal tissues; the results were not similar to those in the serum. We found that IL-6 was significantly higher in the group using a low dose of heat-inactivated *S. thermophilus* MN-002 compared to the other groups. It has been found that enhanced expression of IL-6 in the brain of AD model animals helps enhance microglia to reduce Aβ protein deposition, and the mechanism may be related to the phagocytic ability of microglia activated by IL-6 [36,37]. In addition, our results showed that heat-inactivated *S. thermophilus* MN-002 moderated the expression levels of BDNF (associated with synaptogenesis) and the antioxidant-related enzyme SOD. BDNF plays a crucial role in brain functions, especially cognitive processes, by modulating the growth, survival, differentiation, and homeostasis of neurons and neuroplastic changes [38,39,40]. Research suggests that oxidative stress and excessive accumulation of ROS contribute to cognitive impairment in AD [41]. SOD is an essential enzyme for the conversion of ROS [42]. The significant changes in BDNF and SOD suggest that the regulatory brain function effects of *S. thermophilus* MN-002 may act through modulation of synaptogenesis and modulation of antioxidant-related pathways in the brain, but the exact mechanisms are not yet clear and need to be further verified in our future studies.

These results suggest that the regulatory effects of probiotics and postbiotics on the brain may act through modulation of synaptogenesis and modulation of antioxidant-related pathways in the brain, but the exact mechanisms are not yet clear and need to be further verified in future studies.

## 5. Conclusions

We observed that live and heat-inactivated *S. thermophilus* MN-002 could improve cognitive functions, especially spatial cognition in AD model mice. The improvement could last for three months after the discontinuation of interventions. However, the mechanisms underlying the two forms of the same strain might differ. Live *S. thermophilus* MN-002 acted on cognitive functions in the brain and regulated the composition of the gut microbiota and inflammation. Heat-inactivated *S. thermophilus* MN-002 increased the abundance of *Akkermansia*; the antioxidant capacity of the brain; and the intestinal microbiota’s metabolites, especially propionic acid, thereby improving cognitive functions in APP/PS1 mice. Furthermore, the above changes were more evident in the high-dose groups regardless of whether live or heat-inactivated *S. thermophilus* MN-002 was used; one example was astrogliosis in the brain. These results indicated that different forms and doses of *S. thermophilus* MN-002 could continuously affect the bidirectional communication of the gut–brain axis in different manners, alleviating the cognitive dysfunctions of APP/PS1 mice. Probiotics and postbiotics from *S. thermophilus* MN-002 are an adjunctive prevention and treatment strategy for AD. In this study, we found that alterations in the gut microbiota triggered alterations in its SCFAs, but the specific link between metabolism and cognitive function was not further discussed. Therefore, we will further investigate the metabolic pathway mechanism in a future study.

## Figures and Tables

**Table 1:** Real-time PCR primers.

Target	Sequences
GAPDH	F: 5′-CCT TCC GTG TTC CTA CCC C-3′ R: 5′-GCC CAA GAT GCC CTT CAG T-3′
BDNF	F: 5′-TGG AAC TCG CAA TGC CGA ACT AC-3′ R: 5′-TCC TTA TGA ATC GCC AGC CAA TTC TC-3′
IL-6	F: 5′-GTC ACA GAA GGA GTG GCT A-3′ R: 5′-AGA GAA CAA CAT AAG TCA GAT ACC-3′
TNF-α	F: 5′-CTC TTC AAG GGA CAA GGC TG-3′ R: 5′-CGG ACT CCG CAA AGT CTA AG-3′
Occludin	F: 5′-GCG AGG AGC TGG AGG AGG AC-3′ R: 5′-CGT CGT CTA GTT CTG CCT GTA AGC-3′
ZO-1	F: 5′-GCG AAC AGA AGG AGC GAG AAG AG-3′ R: 5′-GCT TTG CGG GCT GAC TGG AG-3′

**Table 2:** Mean relative abundance of different taxa at phylum level.

	Control (%)	Model (%)	AH (%)	AL (%)	IH (%)	IL (%)
*Actinobacteriota*	0.36	0.70	0.38	0.55	0.59	1.42 *
*Bacteroidota*	29.03	34.19	30.02	53.12 *^#^	45.93	30.60 ^#^
*Campilobacterota*	0.06	0.26	0.08	0.30 *	0.30	0.05
*Deferribacteres*	0.05	0.38 **	0.07 ^#^	0. 10 ^#^	0.10 ^#^	0.04 ^##^
*Firmicutes*	66.40	53.07	63.25	40.04 *	37.30 *	58.58
*Verrumicrobiota*	0.04	1.35 *	0.19	0.19	10.26 **	1.14 *

Compared with control group, * *p* < 0.05, ** *p* < 0.01. Compared with model group, ^#^ *p* < 0.05, ^##^ *p* < 0.01.

**Table 3:** Mean relative abundance of different taxa at genus level.

	Control (%)	Model (%)	AH (%)	AL (%)	IH (%)	IL (%)
*Akkermansia*	0.04	1.10 *	0.19	0.19	10.26 **	1.12
*Bifidobacterium*	0.06	0.26 *	0.02 ^##^	0.09 *^#^	0.08 ^#^	0.24
*Blautia*	0.04	0.10 *	0.12 **	0.02	0.25 **	0.07
*Enterorhabdus*	0.20	0.34	0.20	0.37	0.36	0.68 *
*Helicobacter*	0.05	0.26	0.08	0.35 *	0.30	0.05
*Ileibacterium*	0.03	0.80 **	0. 12	0.16 *	0.15	0.69
*Intestinimonas*	0.07	0.27 **	0.02	0.26 **	0.15	0.05
*Lachnoclostridium*	0.24	0.87 *	0.57	0.58	1.02	0.57
*Lactobacillus*	54.73	18.21 *	41.84	16.57 **	18.63 *	44.08
*Monoglobus*	0.02	0.04 **	0.03	0.03	0.03	0.04
*Mucispirillum*	0.05	0.38 **	0.07 ^#^	0.10 *^#^	0.10^#^	0.04 ^#^
*Negativibacillus*	0.01	0.03 *	0.03	0.02 *	0.02	0.01
*Odoribacter*	0.56	0.55 **	0.41	2.11	1.90 *	0. 35
*Oscillibacter*	0.12	0.18	0.18	0.20 *	0.20	0.06 ^##^
*Parasutterella*	0.08	0.50 **	0.07 ^##^	0.21	0.39	0.10 ^#^
*Parvibacter*	<0.001	0.01	<0.001 ^#^	0.01 *	0.01	0.01 *
*Streptococcus*	0.03	0.02	0.02	0.01	0.18 **	0.01 *
*Turicibacter*	0.07	0.22 *	0.16	0.42 *	1.05	0.70
*Tuzzerella*	0.02	0.04	0.04	0.08 *	0.01 *	0.01 ^##^

Compared with control group, * *p* < 0.05, ** *p* < 0.01. Compared with model group, ^#^ *p* < 0.05, ^##^ *p* < 0.01.

## Data Availability

The data of the 16S rRNA-encoding gene sequences can be found at “https://www.ncbi.nlm.nih.gov/sra/PRJNA1025140 (accessed on 11 October 2023)”. All other supporting data are included within the article.
